# Qualitative analysis of vendor discussions on the procurement of Computerised Physician Order Entry and Clinical Decision Support systems in hospitals

**DOI:** 10.1136/bmjopen-2015-008313

**Published:** 2015-10-26

**Authors:** Kathrin M Cresswell, Lisa Lee, Ann Slee, Jamie Coleman, David W Bates, Aziz Sheikh

**Affiliations:** 1 Usher Institute of Population Health Sciences and Informatics, The University of Edinburgh, Edinburgh, UK; 2 Department of Medical Science and Medical Education, School of Clinical and Experimental Medicine, University of Birmingham, Edgbaston, UK; 3 Department of Medicine, Brigham and Women's Hospital, Harvard Medical School, and the Department of Health Policy and Management, Harvard School of Public Health, Boston MA, USA

**Keywords:** PUBLIC HEALTH, QUALITATIVE RESEARCH, HEALTH SERVICES ADMINISTRATION & MANAGEMENT

## Abstract

**Objectives:**

We studied vendor perspectives about potentially transferable lessons for implementing organisations and national strategies surrounding the procurement of Computerised Physician Order Entry (CPOE)/Clinical Decision Support (CDS) systems in English hospitals.

**Setting:**

Data were collected from digitally audio-recorded discussions from a series of CPOE/CDS vendor round-table discussions held in September 2014 in the UK.

**Participants:**

Nine participants, representing 6 key vendors operating in the UK, attended. The discussions were transcribed verbatim and thematically analysed.

**Results:**

Vendors reported a range of challenges surrounding the procurement and contracting processes of CPOE/CDS systems, including hospitals’ inability to adequately assess their own needs and then select a suitable product, rushed procurement and implementation processes that resulted in difficulties in meaningfully engaging with vendors, as well as challenges relating to contracting leading to ambiguities in implementation roles. Consequently, relationships between system vendors and hospitals were often strained, the vendors attributing this to a lack of hospital management's appreciation of the complexities associated with implementation efforts. Future anticipated challenges included issues surrounding the standardisation of data to enable their aggregation across systems for effective secondary uses, and implementation of data exchange with providers outside the hospital.

**Conclusions:**

Our results indicate that there are significant issues surrounding capacity to procure and optimise CPOE/CDS systems among UK hospitals. There is an urgent need to encourage more synergistic and collaborative working between providers and vendors and for a more centralised support for National Health Service hospitals, which draws on a wider body of experience, including a formalised procurement framework with value-based product specifications.

**Strengths and limitations of this study**

- The vendor perspective provides a unique insight into cross-organisational implementation challenges.
- We have identified important organisational drivers that can ‘make or break’ the implementation journey, long before a formal contract is signed.
- We facilitated an in-depth and open discussion, collecting and analysing the data applying rigorous qualitative methods.
- However, we studied only eight vendors in the English market, and the data collection period was relatively short.

## Introduction

Stimulated by monetary incentives, such as the US Health Information Technology for Economic and Clinical Health (HITECH) Act and the English Integrated Digital Care Technology Fund, the implementation of medication-related Health Information Technologies (HIT) in hospitals has resulted in a recent surge of activity.^1–4^ The underlying hope is that such systems will contribute to enhancing the efficiency, safety and quality of healthcare.^5–8^

In the UK, such systems are commonly referred to as electronic prescribing (or ePrescribing) systems and are associated with functionality surrounding prescribing, decision support at the point of care, transmission of prescribing-related information (eg, when ordering or monitoring), medicines administration, and use of data for secondary uses (eg, quality improvement).^9^ In the USA, commonly associated functionalities include Computerised Physician Order Entry (CPOE) systems that allow ordering of medications tailored to individual patients, and linked Clinical Decision Support (CDS) systems that provide medication-related decision support.

In 2014, approximately 35% of English hospitals had begun implementation of CDS functionality in at least one ward or hospital department, while this figure was over 60% for US hospitals.^10^ ^11^ In England, the previous centralised procurement model for HIT—the National Programme for Information Technology (NPfIT)—has now been replaced by localised decision-making by providers in system choice, in the hope that this will promote local ownership of decision-making and catalyse adoption efforts.^12^ ^13^ There are important lessons the international community can learn from this change in strategic direction, as degrees of political guidance in HIT implementation are deliberated internationally. For example, the USA has adopted a model where hospitals are encouraged to implement accredited technologies stimulated by financial incentives.^4^ However, there are increasing concerns surrounding the governance structures of this approach including the lack of formal evaluation and flexibility, as well as usability and interoperability issues of technologies.^14^

Moreover, early implementing organisations tend to be trailblazers and in many ways may not be representative of the wider hospital community.^5^ ^15^ ^16^ There is therefore a need to build more generalisable knowledge that later implementers can draw on in order to make this journey as smooth as possible. Vendors are a surprisingly underused resource in this respect, although they have an important complementary perspective to that of other stakeholders. While organisational circumstances differ and implementation experience often remains localised, vendors tend to accumulate implementation expertise by moving through a variety of settings and contexts.^17^ ^18^ Their knowledge and experience therefore has the potential to offer important insights into transferable lessons across organisational boundaries. In the English context, some vendors have also gained valuable strategic experiences from operating in the changing political landscape.^19^

The findings reported here explore vendor perspectives surrounding the procurement of CPOE/CDS systems in hospitals in England. In doing so, we build on a substantial body of previous work in primary care and hospital settings, and an ongoing national evaluation of hospital CPOE/CDS systems within the National Health Service (NHS).^3^ ^5^ ^9^ ^13^ ^17^ ^19–28^ We present the results from a series of round-table discussions with vendors that aimed to explore challenges surrounding the procurement of systems, and associated strategies to address these. Our primary research question was: What are the challenges surrounding the procurement of hospital CPOE/CDS systems from the vendors’ perspective and how could these be addressed?

## Materials and methods

### Institutional review board approval

The London City and East Research Ethics Committee classed this work as a service evaluation. Participants were provided with an information sheet offering an overview of the aims of the study and a consent form to be signed prior to the start of the event. The information sheet and consent form described the aims of the study and of how the data would be generated, managed, analysed and used. All participants gave written informed consent to take part.

### Design

This qualitative focus group study was designed to capture the vendors’ perspective on issues, challenges and opportunities arising across the entire system's life cycle from the very early stages at which a hospital plans the introduction of a CPOE/CDS system, right through to the longer term optimisation and integration of the system (box 1). The focus group design was chosen in order to allow exchange of different implementation experiences across systems and contexts.^29^ ^30^

Box 1 **Summary of themes and subthemes identified**

- Limited organisational capabilities in the National Health Service to select, procure and implement systems
  Resources and skills
  Developing and retaining internal capacity to implement
  Senior back-up and drive for implementations
  Rushed implementation timelines
  Understanding current and future processes
- The challenge of procuring a product that suits organisational needs rather than wants
  Differentiating between organisational “wants” and “needs”
  Assessing vendors now and in the future
  The need for value-based product specifications
  Streamlining procurement and contracting processes
- Contracting as the creation of a formal partnership between hospitals and vendors
  Building relationships with vendors before implementation
  Dialogue between vendors and customers
  Conflict resolution and dealing with delays
  Degree of vendor involvement
- Optimising systems and deriving value
  Standardisation
  Secondary uses of data
  Continuing to put the right infrastructure into place
  Integrating and sharing
  Competitive markets and continuing innovation

### Participants

Building on previous work,^25^ a list of 17 key vendors currently actively operating (defined as either having implemented a relevant system or being in active discussions with hospitals planning to implement) in the UK market was drawn up. This shortlist of vendors was then reviewed to include vendors who had either implemented systems in the UK context or in international contexts, excluding vendors who were selling newly developed systems and had no procurement and implementation experience.

This resulted in representatives from 10 organisations being contacted via email and invited to take part. Where possible, contact was made directly with individuals within the organisations whose expertise and knowledge would ensure senior strategic insights could be shared at the event. Where such individuals could not easily be identified or were not available, other suitable representatives were identified by the vendor.

Of the 10 vendors targeted, 8 accepted the invitation of which 6 were represented in the round-table discussions. Of the remaining two vendors from the shortlist, one did not respond to the invitation and another declined to participate. It was agreed that up to a maximum of two representatives from each vendor could attend since relevant expertise and knowledge was in some cases shared across multiple roles within the vendor organisations. As a result, a total of nine participants took part.

### Setting

The event took place in Birmingham, UK in September 2014.^20^ Of the nine delegates taking part, eight were physically present for the event and one joined the round-table discussion from the USA via teleconferencing.

### Data collection and handling

Three areas of interest which reflected the key stages in the implementation and adoption journey undertaken by vendors as well as their vision for the future formed the basis for the discussions (see box 2). These were held as three separate sessions lasting between 60 and 90 min each—one in the morning, and two in the afternoon with either a lunch or coffee break between each. While no data were collected during the breaks, this free time provided participants with the opportunity for further informal deliberation and may have impacted on some of the subsequent discussions.

Box 2 **Key topics discussed**

Topic 1: From conceptualisation to implementation

Key challenges and opportunities for vendors from the early stages of project initiation through to implementation

Topic 2: Go-live and system stabilisation

Vendors' experiences of going live with the system and what lessons can be drawn from roll-outs to date

Topic 3: System optimisation, longer term integration, upgrades and building a vision for the future

Issues relating to short-term and medium-term optimisation, and discussion of the longer term vision and the planning required to ensure service continuity, flexibility and scalability

Sessions were moderated by two members of the research team (ASl and JC). The moderators briefly introduced each topic and provided broad open-ended questions to help initiate discussions. Where possible, moderators sought to let participants guide discussions themselves to ensure that data collected were not constrained by the expectations of the research team.

The audio-recordings were uploaded to a secure server at the University of Edinburgh, and subsequently transcribed by the research team's senior secretary (Rosemary Porteous). The anonymised transcripts were then checked by a member of the research team present at the event (LL).

### Data analysis

We used the qualitative data analysis software package NVivo V.10 for organising the data. The transcripts were uploaded and initially coded along the three topic areas (see box 2) by KMC, an experienced qualitative researcher. We then inductively analysed the categories for recurring themes based on frequency and salience (or agreement among participants), in discussion with LL.^29^ ^30^ After initial analysis, emerging themes and subthemes were refined through discussion within the research team, consisting of experienced social scientists, clinicians and medical informaticists. This discussion was structured around the three main emerging themes surrounding organisational capabilities, procurement and contracting challenges, and system optimisation. It resulted in refinement of subcategories, with individual team members helping to explore clinical and social implications and challenges outlined by participants in more detail.

We continued analysis until we achieved thematic saturation,^31^ the point at which emerging themes were repeatedly identified and no new themes were emerging.

To facilitate analysis, we drew on an analytical framework based on ongoing work,^28^ which depicts the life cycle of technology implementation within healthcare organisations, from conception through implementation towards optimisation. The model consists of the following iterative stages: conceptualisation, project initiation, functional specification, drafting a business case, procurement/tendering, system choice, contracting, preimplementation planning, implementation and optimisation. Conceptualising procurement with this model helped to contextualise findings within the larger implementation journey, as the stages are intimately related and can impact on one another.

## Results

We drew on data from a total of nine participants and analysed three audio-recorded round-table discussions lasting a total of 4.5 h (table 1).

**Table 1:** Characteristics of participants and vendor organisations

	Vendor	Role and background	Gender
Participant 1	A: international implementations, established in the UK	Senior project manager	M
Participant 2	A: international implementations, established in the UK	Senior consultant with NHS nursing background	M
Participant 3	B: international implementations, not established in the UK	Senior manager with a background in sales and marketing, has previously worked at vendor C	M
Participant 4	B: international implementations, established in the UK	Product manager with healthcare IT specialist background	M
Participant 5	C: international implementations, established in the UK, integrated system	Consultant for medical process at with NHS pharmacy background	F
Participant 6	C: international implementations, established in the UK	Clinical strategist with nursing background	M
Participant 7	D: international implementations, established in the UK	Senior manager	M
Participant 8	E: international implementations, not established in the UK	Senior manager, formerly worked for Vendor C	M
Participant 9 (via teleconference)	F: international implementations, not established in the UK	Senior manager leading international implementations of systems	M

F, female; IT, information technology; M, male; NHS, National Health Service.

The following overarching themes were identified (see box 1 for subthemes):

- Limited organisational capabilities in the NHS to select, procure and implement systems;
- The challenge of procuring a product that suits organisational needs rather than wants;
- Contracting as the creation of a formal partnership between hospitals and vendors;
- Optimising systems and deriving value.

These themes will be examined in detail below.

### Limited organisational capabilities in the NHS to select, procure and implement systems

Participants had worked with a number of hospitals and were in broad agreement that, from their experience, there was a lack of resources to support the appropriate and effective selection, procurement and implementation of CDS/CPOE systems in NHS hospitals. In this context, skills shortages in hospitals where systems were deployed were particularly frequently mentioned, including limited expertise in business change, benchmarking, product testing, contracting, project management and service improvement. In line with this, the staffing of the implementation teams in hospitals was viewed as inadequate in many cases, with hospital teams lacking the right multidisciplinary combination of knowledge surrounding clinical processes, project management and software delivery processes.

You're buying a software product […] and therefore you need to understand how software is made, released, tested, accepted and all those things that we know as a software company we do. And if the project manager has no understanding of those sequences, especially where they […] are releasing new functionality […] almost quarterly, that all has to be factored in. Participant 8, Vendor E

However, it was explained that organisations needed to develop internal capacity to implement systems, as well as to retain skilled staff. Vendors observed that many hospitals employed external consultants on short-term contracts as project managers, but these individuals tended to lack inside organisational knowledge and took their skills with them when they left.

We're getting people coming from outside organisations…but you haven't got a clue about what the organisation is like and you spend six months learning what the organisation is doing today. Never mind what it needs to do tomorrow. And I think that's part of the problem…you need to be growing that expertise in-house as part of your work… Participant 1, Vendor A

In addition, senior commitment from lead physicians and/or chief executives to support implementation efforts was often lacking and this hampered progress as decisions were not made and visions of future states were not effectively communicated organisation-wide.

In terms of monetary resources, the current financial payment profile and milestone structure in the NHS were viewed as being governed by an outcome-based funding principle, where project milestones were seen to be determined by availability (or lack) of money. For example, vendors were paid for milestones such as ‘going-live’, as opposed to receiving reimbursement for important strategic planning activities that preceded the actual go-live event. As a result the focus shifted from planning to outcomes, which was not always seen to be beneficial for implementations.

Just anything to do with when the money is coming into the [hospital] needs to be decoupled from the broader strategy. Because otherwise we get the perverse behavior “everything needs to be done by this time because that's when we're going to get the money […]”. We shouldn't have money for go-live; we shouldn't be paid a lot of money for me turning up on site because that's a good milestone… Participant 2, Vendor A

The impact of financial resources on implementation progress was also referred to on a national scale. Although the NHS’ Technology Fund was viewed to stimulate the initial phases of implementations, it was seen as limited in helping hospitals to resource further roll-out and optimisation as it consisted of relatively limited funding over a finite period of time.

As a result of financially driven milestones, implementations were regularly seen as being rushed with hospitals wanting to implement complex functionality too early. Vendors, on the other hand, felt that implementation timelines needed to be more considered, beginning with implementing relatively straightforward functionality in less complex areas in order to help organisations get used to the basic changes brought about by the new system in relatively stable environments. The roll-out was then ideally governed by implementing in areas that were somehow linked to each other. In this context, the notion of a ‘pilot’ was viewed as unhelpful for the implementation process as systems were not, as in conventional pilot projects tested and then modified, but implemented gradually throughout the organisation.

I wouldn't call it a pilot phase, for me this is about the first wards to go live, not pilot because pilot is like testing something that might not actually be used and I like not to use the word pilot. Participant 2, Vendor A

This strategy would also help hospitals to learn from areas that went live first. Incorporating these lessons was seen as a crucial part of the implementation process, although frequently overlooked.

They [hospitals] want to go live on the system with all the bells and whistles there and then, which will therefore risk future adoptions. Users are going to grab that paper chart because they know how to use that paper chart […]. Whereas I think it's much better from my experience to go-live with something which is relatively simple, potentially replicates paper, so you roll out this advanced functionality be it drug checking, adverse drug reaction forms that kind of stuff after the initial adoption stage has pitted out. Participant 5, Vendor C

A lack of considered in-depth planning was also observed in relation to an often inadequate understanding of current and future business and work processes. As a result, managers in hospitals were unaware of the way systems were actually used and existing processes were modified to accommodate systems.

I'm always surprised to see the number of workarounds that hospitals will put into place. And the actual project management […] and the IT staff that we would work directly with actually have no idea of what's actually happening on the ward. And a lot of times that affects them when they go through things like regular system updates and things like that. They're not testing their own processes because they don't understand their own processes. Participant 9, Vendor F

Mapping of future processes and ensuring that these were adhered to, was viewed as important, as this would help to ensure that best practice guidelines would be followed and systems would be used effectively.

### Procuring a product that suits organisational needs rather than wants

Procuring a product that suited and addressed organisational needs was seen by vendors as a key decision. A distinction needed to be made between what organisational managers *thought* they needed (which was often different from they *actually* needed) and what decision makers wanted.

Understanding the organisation's need and procuring a product based on this was also seen as the best way to ensure that functionalities were effectively adopted. In this context, it was reiterated that complex functionalities (which were often on wish lists of hospitals) were not necessarily suitable for all organisations, particularly if they were implemented too early on in the process.

What I want is everything that gets talked about up to the point where you go live and you suddenly realise that you don't want all of these. What you need is this, and this is the difference between giving everything that sparkles and is bright on day one or just giving a system that works on day one. Participant 2, Vendor A

Before formal procurement had started, when deciding on a system, frequent contact with potential vendors and other organisations that were further on in the implementation journey was seen as helpful. Scripted system demonstrations (where organisations determine in advance what they want vendors to demonstrate) were viewed of limited value, as these failed to take account of and reflect providers organisational circumstances. The need for a more comprehensive evaluation of vendors during requirement evaluation was therefore highlighted.

Scripted demos are the norm in most places and they can actually end up in a bias. Having a proper business case model, you should be going in assessing the suppliers in some format that you can score, because otherwise you end up with a mixed matrix of scores and you've got to question how valid is that score at the end of the day. Is it subjective or objective? Participant 7, Vendor D

As part of the assessment of vendors, an assessment of future functionality was stated to be necessary in order to determine the compatibility of the product and the organisation. This was, however, viewed as being complicated by the current immaturity of the implementation landscape and existing systems.

Participants further argued that product specifications, a common feature of procurements, were constraining and often too granular to reflect realistic product assessments. Although the notion of output-based specifications (OBS) was welcomed over the use of functional specifications in helping to move away from technical features towards process improvement; participants argued that value-based outputs that allowed vendors to understand reasons behind technological requests were still lacking in current procurements.

I think one of the things that we need to be trailing towards is value based statements. So why are you doing it, why do you want the answer to this question? So it's “I want to do something because” and we don't have that in either functional specs [specifications] or OBS’ at the moment… Participant 1, Vendor A

The issues outlined above could partly be addressed by streamlining procurement and contracting processes, as an overall framework for procurement was still lacking. Participants recommended that this framework should include an organisational readiness assessment before procurement to ensure that necessary resources and skills are available, and giving vendors time and information about local organisational processes before system demonstrations to make these more locally relevant.

### Contracting as the creation of a formal partnership between hospitals and vendors

Vendors highlighted how building relationships with hospitals well before implementation was important in order to explore together what system would be best suited to the needs of the organisation. Such relationship building, although time-consuming for both sides, could help hospitals to avoid purchasing systems that were not suitable for them and help vendors understand expectations. It was stated that this dialogue between customers and vendors should ideally happen before the tendering phase, as otherwise the discussion would be commercially constrained.

Contracting was ideally viewed as the creation of a formal partnership with hospitals. However, this was not always the case, with many suggesting that the establishment of formal contracts with the NHS felt more like a risk transfer from hospitals to vendors, governed by inflated expectations and penalisation for missing deadlines.

The ideal contract is where you have a partnership, to use that cliché, and that the contract sits on the shelf and away you go with the implementation plan and the thing goes forward. There's a lack of appreciation sometimes as why, from a commercial point of view, that it needs to be a balanced contract. Invariably it's a risk transfer completely to the commercial side; it's not a risk share… Participant 7, Vendor D

Delays on the hospital side were often felt to be due to bureaucratic structures that hampered speedy decision-making and competing demands on senior decision makers’ time. This was not always seen as adequately considered and documented, with many hospitals ‘blaming’ vendors for implementation delays.

If you have a clinical problem that needs to be solved by a clinical advisory group or some other steering group, because we need to make a definitive change or step change in policy or procedure, that can take weeks and weeks and weeks. Because that clinical advisory group or drug and therapeutics team may not meet more than every two months, so those kind of things cause delays. Participant 2, Vendor A

Delays on the vendor side related mainly to processing change requests for customisation, but the complexity of these was often not sufficiently appreciated by the NHS hospitals. Vendors noted that customisation was not desired as it would make their product less generic and therefore harder to implement across other sites. Nevertheless, the speed at which requests were attended to was governed by external demands such as regulatory mandates, which meant that individual change requests for customisation that were not crucial for patient safety were often a low priority. Also, the potential impact of change requests on different parts of the system meant that they had to be subjected to lengthy testing procedures.

So you do get some customers who say “oh can't you not just change this” and you get great problems in managing client satisfaction when you say “yeah it might be 12 months from now before you get that unless it's a clinical safety issue”, which most of us tend to respond to right away. Participant 7, Vendor D

It was generally felt that contracts were only referred to when ‘things went wrong’ and vendors highlighted that therefore important contractual components included processes surrounding conflict resolution and ways to deal with implementation delays. Contracts further needed to include an outline of the involvement of vendors during roll-out, but hospitals frequently underestimated the change management assistance and expertise vendors could offer. Although vendor support was dependent on existing organisational structures, needs and resources, it was seen to be frequently underestimated in most contracts.

Well they [hospitals] never ask for enough [support] in my opinion. Never ask for enough. They completely underestimate what value the supplier can bring to them. And they always ask for it post-contract of course. Participant 8, Vendor E

### Optimising systems and deriving value

Secondary uses of data were seen as a major way to optimise systems and derive value from the investments made. However, it was acknowledged that in order to achieve maximum returns for all stakeholders and enable meaningful aggregation, data across systems would need to be standardised.

I think we've got a responsibility as suppliers to standardise the data that we have amongst our clients. I kind of think we're not there yet speaking from our point of view but it's going towards that standardisation… Participant 5, Vendor C

Participants also highlighted that effectively drawing on secondary uses of data would take time, particularly in hospitals that were slow to adopt systems (as adoption was a prerequisite for deriving value through secondary uses of data). Continuing to put the right infrastructure into place was therefore seen as an intermediate goal for vendors in order to build a solid platform from which to optimise uses in the future.

Integrating systems and sharing data across care settings, particularly between general practitioners (GPs), social care providers, pharmacies and hospitals, and with patients was seen as another major future development.

It's getting information back to GP systems. It means getting electronic transfer of prescriptions sorted out to local chemists. It means sharing information with social carers so they can see what Mable has to have three times a day and what the tablets look like […] That's probably the next big step in my mind… Participant 2, Vendor A

In order to achieve visions of an integrated healthcare system and derive value through secondary uses of data, vendors stressed the importance of competitive markets and continuing innovation. But these were seen to be heavily influenced by political decisions and funding restrictions. England's NPfIT^12^ ^13^ was given as an example where political decisions had stifled healthy commercial competition and inhibited effective team working between vendors and hospitals through centrally governed contracts.

I'd say the one big vision thing that we probably all agree with is […] a continued, funded, vibrant, competitive market place. Because the danger is health services can do another National Programme. We'll go into another 10 year hiatus and God knows what else. Participant 7, Vendor D

Another potential inhibitor of progress was the commercial capacity to deliver systems in a time of increasing interest by hospitals and acceleration by the English Technology Fund. As a result, vendors found themselves ‘cherry picking’ hospitals for implementation as they did not have the capacity to deliver on all tenders.

## Discussion

We found that the CPOE/CDS vendor perspective provides important insights into cross-organisational implementation challenges. We identified important organisational drivers that can ‘make or break’ the implementation journey, long before any formal contract is signed. Rushing of procurement processes can result in a lack of appropriate appreciation of organisational needs and meaningful engagement with different vendors, resulting in acquisition of a product ill-suited to the context of deployment. Once a contract is signed, constructive dialogue between vendors and customers can be inhibited by a lack of clarity surrounding implementation roles. Vendors represent a valuable source of expertise that is often underappreciated by hospitals implementing clinical information technology (IT) systems. Important future challenges surrounding system optimisation include standardisation of data so that these can be aggregated across systems for meaningful secondary uses, and implementation of data exchange with organisations outside the hospital.

Our results indicate that a certain degree of centralisation of procurement and contracting processes may, in a UK context, help to address the challenges identified. We have summarised these in box 3. The need for a more formalised procurement framework was frequently highlighted by our participants, as currently English hospitals appear to lack expertise in this area. It seems that the ‘post-NPfIT autonomy’ surrounding procurement has come at a price—while hospitals can now choose software vendors, they find it difficult to do this in a considered way and there is a shortage of expertise and experience in negotiating what are often very sizeable contracts. Some form of federal support therefore appears to be needed, but achieving a balance between governmental assistance and perceived central control will be difficult.^13^ A potential strategy could take a shape similar to the early stages of the Meaningful Use Criteria in the USA,^24^ being based around value-based product specifications, as suggested by our participants. The English Department of Health's National Information Board framework has begun to tackle some of these issues, including establishing a digital support service to help organisations comply with national digital standards.^32^ This also considers the organisation's digital maturity. Here, it will be important to build on existing commercial and procurement guidance to include considerations surrounding interoperability to ensure effective future exchange of information between organisations.^33^ As our work indicates, this can only be achieved if some centralised procurement guidance surrounding system accreditation exists, as individual organisations are likely to seek short-term value (while setting up interoperable systems form the start tends to be more costly in the short term). We have summarised some strategies for organisations to consider in relation to vendor and product selection in box 4.

Box 3 **Potential ways to address cross-organisational procurement challenges through increased central guidance**

- Increasing standards surrounding procurement and contracting processes (building on existing purchasing guidance through centralised system accreditation and standards)
- More considered implementation timelines (locally and nationally)
- A central resource helping the National Health Service to develop skills to procure and implement systems effectively
- Guidance to support product choice through devising functional, output-based, and value-based specifications
- Ensuring that commercial markets remain competitive and no monopolies emerge to ensure continuing innovation
- Continued federal funding to support procurement, implementation, and optimisation
- Guidance surrounding the standardisation systems to allow integration and sharing on larger scales in the future

Box 4 **Strategies for organisations to consider in relation to vendor and product selection**

- Taking time throughout procurement processes to assess organisational needs and base
- product/vendor selection on this need
- Meaningful long-term engagement with different vendors and assessment of a range of products
- Harnessing implementation expertise of vendors and making this explicit in contractual arrangements
- Choosing products that are likely to be able to cope with future challenges surrounding secondary uses of data and interoperability

In light of existing commercial sensitivities surrounding contracts and procurement, and an associated under-representation of vendor perspectives, this study has helped to explore procurement-related insights and potentially transferable lessons. However, this work also has a number of limitations. We studied eight vendors, resulting in a relatively small number of participants representing a limited amount of (albeit key) organisations operating in the UK, and the data collection period was relatively short. This may have resulted in potentially limited transferability of findings. Nevertheless, we have brought together high-level representatives from the key CPOE/CDS system vendors in the English market, allowing a very rich, interactive and open discussion (which would have been difficult with a greater number of participants). By recording it and coding emerging concepts, we were able to identify a number of what we believe are important lessons, although these need to be interpreted within the English context. For example, the dominance of Epic in the North American market has been the subject of recent discussions, with fears that one commercial company will have a monopoly—a similar situation already encountered in the English NPfIT.^34^ Participants were acutely aware of the potential pitfalls of such a monopoly, fearing that it can hinder commercial competition and stifle innovation.^35^ Most emerging themes were common among participants, with no divergent opinions on the main issues. There were, however, some minor disagreements in relation to specifics, particularly regarding the degree of standardisation of contracts between healthcare organisations and vendors, the nature of vendor involvement in actual implementation-related activities, and the commercial capacity to deliver desired functionality.

Some of our findings mirror existing evidence in relation to the significance of senior management and physician buy-in to support implementations;^36^ ^37^ adequate financial resources to finance and incentivise initiatives;^38^ ^39^ and work process redesign to realise benefits associated with technologies.^40–42^ The significant potential of secondary uses of data from systems has also been previously highlighted. Our work adds important perspectives surrounding procurement and contracting processes and how these need to suit individual organisations while also ensuring that wider national needs are met such as larger scale information exchange. Such views may be missing in current managerial discourses, as these are often focused on localised organisational change. Important areas in this context include the creation of formal partnerships between hospitals and vendors; and the optimisation of systems and the derivation of data. Future work should examine these issues in more detail, particularly in relation to a potential alignment of organisational and vendor priorities, and determine whether they have international relevance or reflect England's individual circumstances.

Our results also suggest that the UK healthcare sector is lagging behind other industries in relation to forging effective relationships with commercial vendors. It has been argued that this is partly due to a disconnect between the organisational purchasing department and decision makers. Consequently, there tends to be a drive to get the best deal at the lowest price resulting in a lack of trust between vendors and hospitals. These strained relationships need to be more effectively managed by drawing on more sophisticated strategies from other industries.^43^ Examples may include proactive approaches that allow cooperative partnerships between hospitals and vendors, as well as robust and more coordinated procurement strategies (as discussed above) with associated process evaluations.^44^ ^45^

## Conclusions

The experiences of procuring CPOE/CDS systems in England's NHS illustrate how the political environment surrounding implementations can affect relationships on the ground. As implementing organisations are now required to procure their own systems, progress has been hampered by limited organisational capacity to select suitable products. More centralised procurement support has the potential to be beneficial, but the balance between ‘too much’ and ‘too little’ government involvement will be hard to achieve.
